# Mutation screening in the *FBN1* gene responsible for Marfan syndrome and related disorder in Chinese families

**DOI:** 10.1002/mgg3.594

**Published:** 2019-03-05

**Authors:** Bo Gong, Lan Yang, Qingwei Wang, Zimeng Ye, Xiaoxin Guo, Chen Yang, Fang Hao, Yi Shi, Yi Huang, Chao Qu, Zhenglin Yang

**Affiliations:** ^1^ Sichuan Provincial Key Laboratory for Disease Gene Study and Department of Laboratory Medicine Sichuan Academy of Medical Sciences and Sichuan Provincial People’s Hospital, School of Medicine, University of Electronic Science and Technology of China Chengdu, Sichuan China; ^2^ Institute of Chengdu Biology Sichuan Translational Medicine Hospital, Chinese Academy of Sciences Chengdu, Sichuan China; ^3^ Department of Ophthalmology Sichuan Academy of Medical Sciences and Sichuan Provincial People’s Hospital, School of Medicine, University of Electronic Science and Technology of China Chengdu, Sichuan China; ^4^ School of Clinic Medicine Southwest Medical University Luzhou, Sichuan China; ^5^ School of Medicine, Dentistry and Health Sciences The University of Melbourne Melbourne Victoria Australia

**Keywords:** *fibrillin‐1* (*FBN1*), heterozygous mutation, Marfan syndrome (MFS), targeted next‐generation sequencing (NGS)

## Abstract

**Background:**

Previous studies showed that the *fibrillin‐1* gene (*FBN1*) is responsible for Marfan sydrome (MFS) pathogenesis. This study is conducted to screen for mutations in the *FBN1* gene in Chinese families with MFS.

**Methods:**

Eight families with MFS and related disorder were recruited in this study. All available family members underwent complete physical, ophthalmic, and cardiovascular examination. Mutation screening was performed using targeted next‐generation sequencing. Candidate variants were amplified by polymerase chain reaction and verified by direct Sanger sequencing.

**Results:**

Four novel heterozygous mutations in *FBN1*, including c.2861G>T (p.R954L), c.4087G>A (p.D1363N), c.4987T>G (p.C1663G), and c.5032T>G (p.Y1678D), as well as four known mutations, c.3617G>A (p.G1206D), c.4460A>G (p.D1487G), c.4588C>T (p.R1530C), and c.718C>T (p.R240C) were identified. Affected patients from each family were found to carry one of the mutations, whereas the unaffected members and 1,086 normal controls were not. Each mutation was found to be cosegregated with MFS phenotype and related disorder in each family. Multiple sequence alignment of the human fibrillin‐1 protein showed that these mutations occurred in a highly conserved region among different species.

**Conclusions:**

Eight *FBN1* mutations were identified in Chinese families with MFS and related disorder. These data expands *FBN1 *mutation spectrum and further emphasizes the role of *FBN1* in the pathogenesis of MFS.

## 1 INTRODUCTION

Marfan syndrome (MFS) is an autosomal dominant hereditary and multiple‐systemic disease, mainly involving the ocular, skeletal, and cardiovascular systems (Judge & Dietz, 2005). It has large clinical variability (both within and between families) and genetic heterogeneity (Dietz et al., 1995; Faivre et al., 2015). According to the revised Ghent criteria for MFS (Radonic et al., 2011), the cardiovascular and ocular manifestations, including aortic root aneurysm and ectopia lentis, are sufficient for the unequivocal diagnosis of MFS with or without a positive family history.

In 1991, heterozygous mutations in the *fibrillin‐1* gene (*FBN1*, OMIM: 134,797) coding for fibrillin‐1 were reported to cause MFS (Tynan et al., 1993). *FBN1* mutations have been found in >90% of MFS. At present, more than 3,000 mutations in the *FBN1* gene have been identified in relation to MFS (Xiao et al., 2017). Most mutations are unique in each MFS family, and only approximately 10% of mutations are recurrent among different families (Dong et al., 2012). The *FBN1* gene, located at chromosome 15q‐21.1, is comprised of 65 exons spanning 235 kb of genomic DNA. It encodes a secreted 350‐kDa glycoprotein (Sakai, 1986) that is highly conserved among different species. Fibrillin‐1 is a large modular glycoprotein that assembles to form 10‐ to 12‐nm microfibrils in the extracellular matrix (Pereira et al., 1993). These microfibrils provide force‐bearing structural support in elastic and nonelastic connective tissue throughout the body. The intracellular dominant negative or haploinsufficiency mutations of FBN1 are the main pathogenic mechanism of MFS (Dietz et al., 1993; Judge & Dietz, 2005; Matyas et al., 2007). Therefore, molecular genetic testing plays a prominent role in the diagnosis of MFS, particularly for children or suspicious patients (Radonic et al., 2011).

In this study, we characterized the clinical manifestations and investigated the molecular basis of eight Chinese families with MFS, to screen for mutations that cause MFS using targeted next‐generation sequencing (NGS) method and potentially promote the understanding of pathogenesis of MFS.

## 2 MATERIALS AND METHODS

### 2.1 Ethic committee statement and subject recruitment

**Figure 1:**
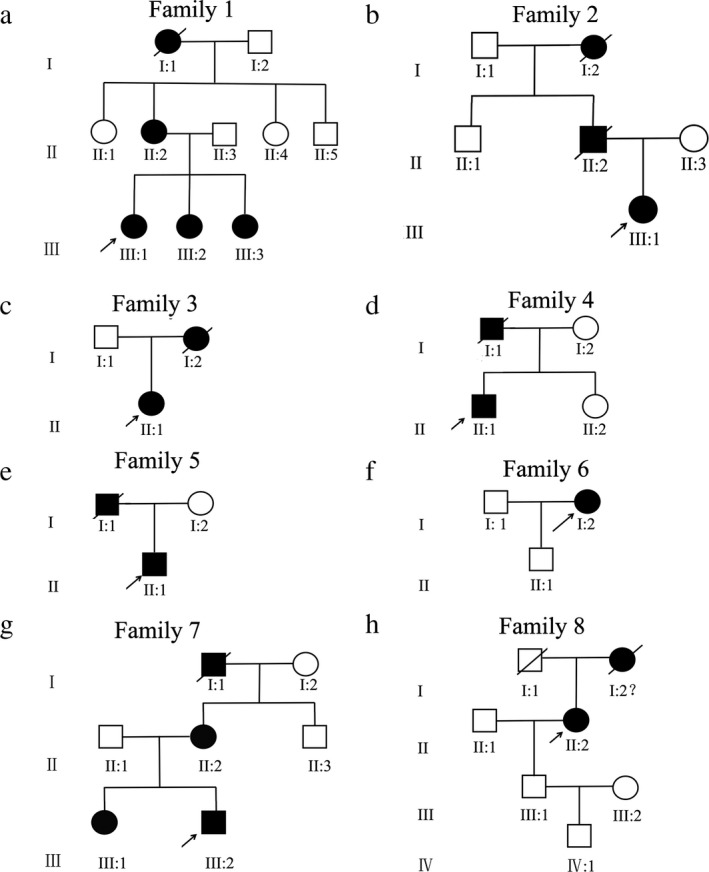
Eight families with MFS and related disorder were recruited in this study. Squares represent males; circles represent females; solid symbols indicate affected patients; open symbols indicate unaffected subjects; and arrow indicates the proband in this family

The ethic committee of Sichuan Provincial People's Hospital approved the project and investigators followed the principles of the Declaration of Helsinki. Eight nonconsanguineous families with MFS were recruited from Sichuan Provincial People's Hospital (Figure 1) in this study. Informed consent was obtained from each patient and their related families before genetic testing. According to the revised Ghent criteria (Radonic et al., 2011), each proband in the eight families was diagnosed with MFS and related disorder. All available members of eight families underwent complete physical, cardiovascular, and ophthalmological examinations. A total of 1,086 ethnically matched, unrelated, and normal healthy individuals were recruited from Sichuan Provincial People's Hospital. These control individuals, also underwent the same examinations, had no medical history associated with any related diseases.

#### 2.1.1 Mutation screening

Genomic DNA samples were extracted from peripheral blood using a Qiagen FlexiGene DNA kit (Qiagen, Duesseldorf, Germany). These DNA samples from II:2, II:4, III:1, III:2, and III:3 in family 1, I:1, II:1, II:3, and III:1 in family 2, I:1, I:2, and II:1 in family 3, I:1, I:2, II:1, and II:2 in family 4, I:2 and II:1 in family 5, I:1, I:2, and II:1 in family 6, II:1, II:2, II:3, III:1, and III:2 in family 7, II:1 and II:2 in family 8, were analyzed by targeted NGS, respectively. As reported in previous study (Xiao et al., 2017), a custom‐designed gene panel, synthesized by the Agilent Sure‐Select Target Enrichment technique (Kangso Institute, Beijing, China), was used to capture the coding regions from 331 genes, including their exons and exon–intron boundaries (1.285 M bp in total). The detected variants were annotated and filtered based on public and in‐house databases: (i) variants within intergenic, intronic, and UTR regions and synonymous mutations were excluded from downstream analysis; and (ii) variants in dbSNP138 (http://www.ncbi.nlm.nih.gov/projects/SNP/), 1,000 Genomes Project (ftp://ftp.1000genomes.ebi.ac.uk/vol1/ftp), YH Database (http://yh.genomics.org.cn/), and HapMap Project (ftp://ftp.ncbi.nlm.nih.gov/hapmap) were excluded. Heterozygous variations of genes with autosomal dominant heredity were regarded as likely causative variations. We performed validation and parental origin analysis for these identified variations using conventional Sanger sequencing method. The causative mutations were confirmed according to parental origin of the variations and clinical features of the patients. The sequences of above variations sites in *FBN1* (NM_000138.4/NP_000129.3) were obtained from GenBank and amplified by polymerase chain reaction (PCR) using the primers in previous study (Xiao et al., 2017). Amplified PCR products were purified and sequenced directly (BigDye Terminators Sequencing kit) with an Automated Genetic Analysis system 3,130 (both from Applied Biosystems; Thermo Fisher Scientific, Inc.). At last, the possible damaging effects of the mutation on the structure and function were predicted using SIFT, PolyPhen‐2, Mutationtaster, and ACMG classification.

## 3 RESULTS

### 3.1 Clinical findings

All the eight families with MFS were recruited from Sichuan Province (Figure 1). Clinical information of patients was summarized in Table 1. Affected patients from these families exhibited similarly clinical symptoms of MFS, including aortic aneurysm, ectopia lentis, myopia, strabismus, arachnodactyly, flat feet, and so on. All the healthy family members had no features of MFS.

**Table 1:** Clinical information of patients in the families with MFS

Family	Patient	Age (years)	Ocular	Cardiovascular	Skeletal
1	II:2	48	EL, M, S	‐	AR, PC, Sco
	III:1^a^	24	EL, M, S	AA (45 mm)	AR, PC, Sco
	III:2	17	EL, M, S	ARD (33 mm)	AR, PC, Sco
	III:3	10	EL, M, S	ARD (32 mm)	AR, PC, Sco
2	III:1^a^	3	EL, M, S	‐	AR, PC, F
3	II:1^a^	10	EL, M, S	‐	AR, PC
4	II:1^a^	18	EL, M, S	AA (treated)	AR
5	II:1^a^	3	EL, M, S	‐	‐
6	I:2^a^	54	EL, M, S	‐	AR, PC, F
7	II:2	42	EL, M, S	‐	‐
	III:1	9	EL, M, S	‐	‐
	III:2^a^	7	EL, M, S	‐	‐
8	II:2^a^	51	EL, M, S	AA (44 mm)	AR, PC, Sco, HD

Note

‐: not available; EL: ectopia lentis; M: high myopia >6.0D in both eyes; S: strabismus; AA: aortic aneurysm; ARD: aortic root diameter; AR: arachnodactyly; Sco: scoliosis, PE: pectus excavatum; PC: pectus carinatum, F: flatfeet; HD: hindfoot deformity.

a Proband.

**Figure 2:**
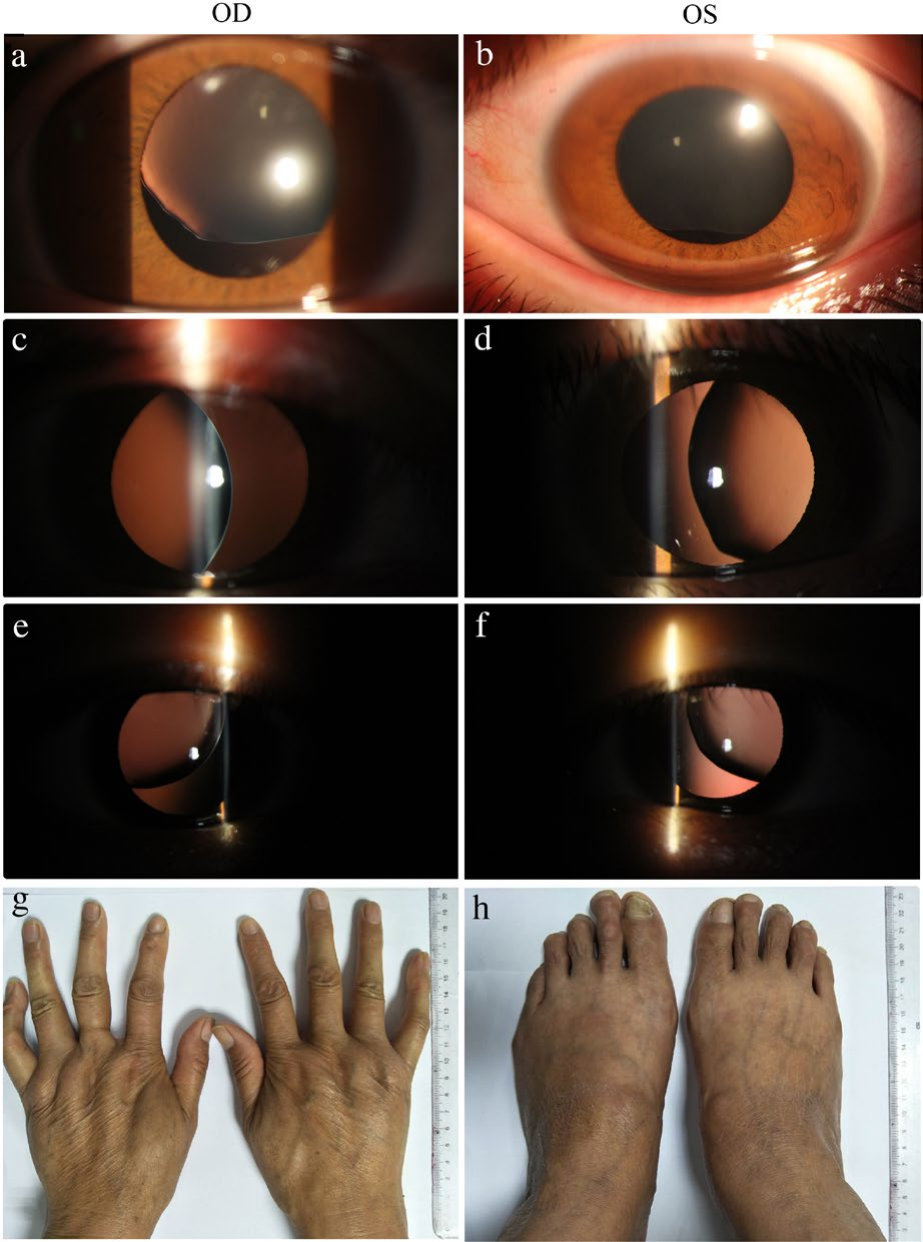
Clinical features of MFS patients. (a and b) Slit lamp photograph showed that both eyes of the proband in family 1 (III:1) had ectopia lentis with lens superior deviation; (c and d) lens temporal deviation occurred in both eyes of the proband's young sister (III:2) in family 1; (e and f) lens temporal‑superior dislocation occurred in both eyes of the proband's younger sister (III:3) in family 1; (g and h) the proband in family 8 (II:2) had long fingers and flat feet. OS, oculus sinister (left eye); OD, oculus dexter (right eye)

In family 1 (Figure 1a), slit lamp photograph showed that two eyes of the proband had ectopia lentis with lens superior deviation (Figure 2a‐b), whereas lens temporal deviation (III:2, Figure 2c‐d) and temporal‐superior dislocation (III:3, Figure 2e‐f) were observed in both eyes of the proband's young sisters, respectively. The affected patients of family 2 (Figure 1b), family 4 (Figure 1d), family 5 (Figure 1e), and family 6 (Figure 1f) exhibited similarly clinical symptoms including ectopia lentis, myopia, strabismus, arachnodactyly, flat feet, and so on, but no abnormality in cardiovascular system. Among of them, the father and grandmother of the proband of family 2 suffered from a classical MFS and died of a heart attack at the age of 42 and 66 years, respectively. The affected patients in family 3 (Figure 1c) and family 7 (Figure 1g) merely presented with abnormality of ocular system. In family 7, the mother and elder sister of the proband also suffered from ectopia lentis and high myopia. In family 8 (Figure 1h), the proband had the facial and skeletal features, involving long fingers and flat feet (Figure 2g‐h). Her mother was highly suspected of MFS and died of a heart attack when she was at age of 50 years.

### 3.2 Mutation screening of *FBN1*

**Figure 3:**
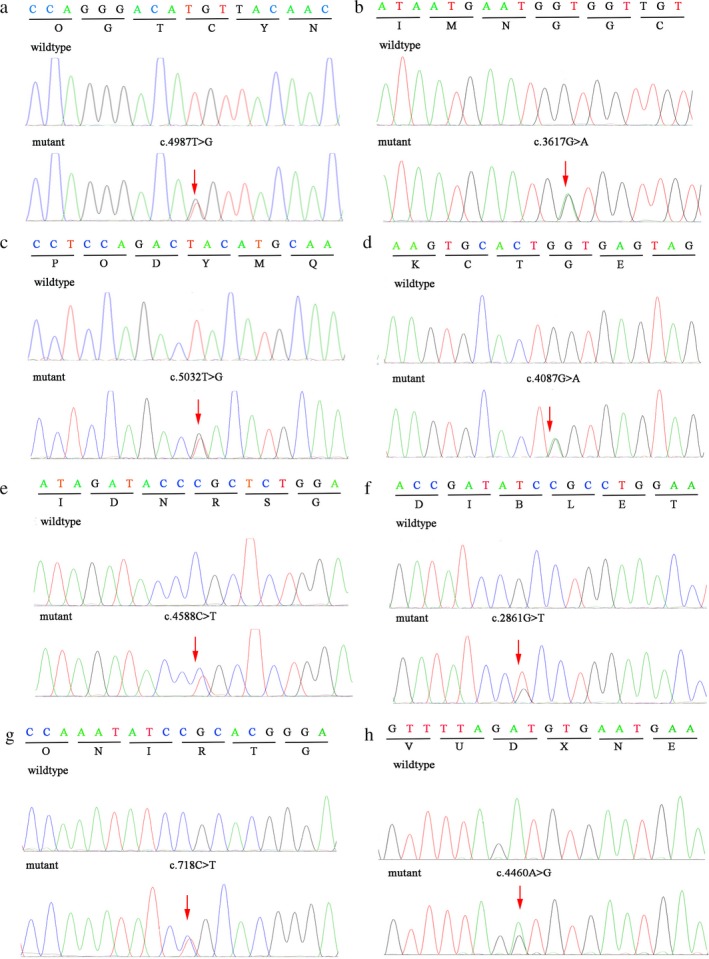
*FBN1 *mutations were identified in eight families with MFS. Each mutation was validated using Sanger sequencing. (a) The mutation c.4987T>G was detected in the patients of family 1; (b) the mutation c.3617G>A was detected in the patients of family 2; (c) the mutation c.5032T>G was detected in the patients of family 3; (d) the mutation c.4087G>A was detected in the patients of family 4; (e) the mutation c.4588C>T was detected in the patients of family 5; (f) the mutation c.2861G>T was detected in the patients of family 6; (g) the mutation c.718C>T was detected in the patients of family 7; (h) the mutation c.4460A>G was detected in the patients of family 8

The quality and reliability of targeted NGS data were evaluated based on the percentage of readable bases and the coverage depth in the targeted region, to ensure complete sequencing coverage of all coding regions in candidate genes. The coverage depth was up to 200×, with 100% of bases being readable in coding regions. On average, 574 variations within the 331 genes were covered in the analyzed samples. Under the autosomal dominant model, the filtered data was narrowed down to a pathogenic heterozygous variant in each family. Each variant, considered as causative candidate and pathogenic mutation, was further validated using Sanger sequencing method in other family members and 1,086 normal controls. Finally, we confirmed eight heterozygous mutations including four novel mutations of the *FBN1 *gene in the patients, including c.2861G>T (p.R954L), c.4087G>A (p.D1363N), c.4987T>G (p.C1663G) and c.5032T>G (p.Y1678D), as well as four known mutations, c.3617G>A (p.G1206D), c.4460A>G (p.D1487G), c.4588C>T (p.R1530C), and c.718C>T (p.R240C) (Figure 3 and Table 2). These mutations were absent in the unaffected members and the other 1,086 normal controls. Therefore, these mutations were cosegregated with the phenotype in each family.

**Table 2:** *FBN1 *mutations of all families with MFS

Family	AA Substitutions	Mutations	Exons	Protein Domains	SIFT	Polyphen2	Mutationtuster	ACMG classification
1	C1663G	**c.4987T>G**	41	cbEGF‐like NO.24	Deleterious	Probably damaging	Disease causing	Likely pathogenic
2	G1206D	**c.3617G>A**	30	cbEGF‐like NO.15	Deleterious	Probably damaging	Disease causing	Uncertain significance
3	Y1678D	**c.5032T>G**	41	cbEGF‐like NO.24	Deleterious	Probably damaging	Disease causing	Uncertain significance
4	D1363N	**c.4087G>A**	33	cbEGF‐like NO.19	Deleterious	Probably damaging	Disease causing	Uncertain significance
5	R1530C	**c.4588C>T**	38	Cysteine domain NO.04	Deleterious	Probably damaging	Disease causing	Likely pathogenic
6	R954L	**c.2861G>T**	25	Cysteine domain NO.03	Deleterious	Probably damaging	Disease causing	Uncertain significance
7	R240C	**c.718C>T**	7	Hybird module NO.01	Deleterious	Probably damaging	Disease causing	Likely pathogenic
8	D1487G	**c.4460A>G**	37	cbEGF‐like NO.22	Deleterious	Probably damaging	Disease causing	Uncertain significance

Note

+: heterozygous missense mutation; Deleterious: possibly suffered from MFS; probably damaging: probably suffered from MFS; disease causing: possibly suffered from MFS; disease causing automatic: probably suffered from MFS.

**Figure 4:**
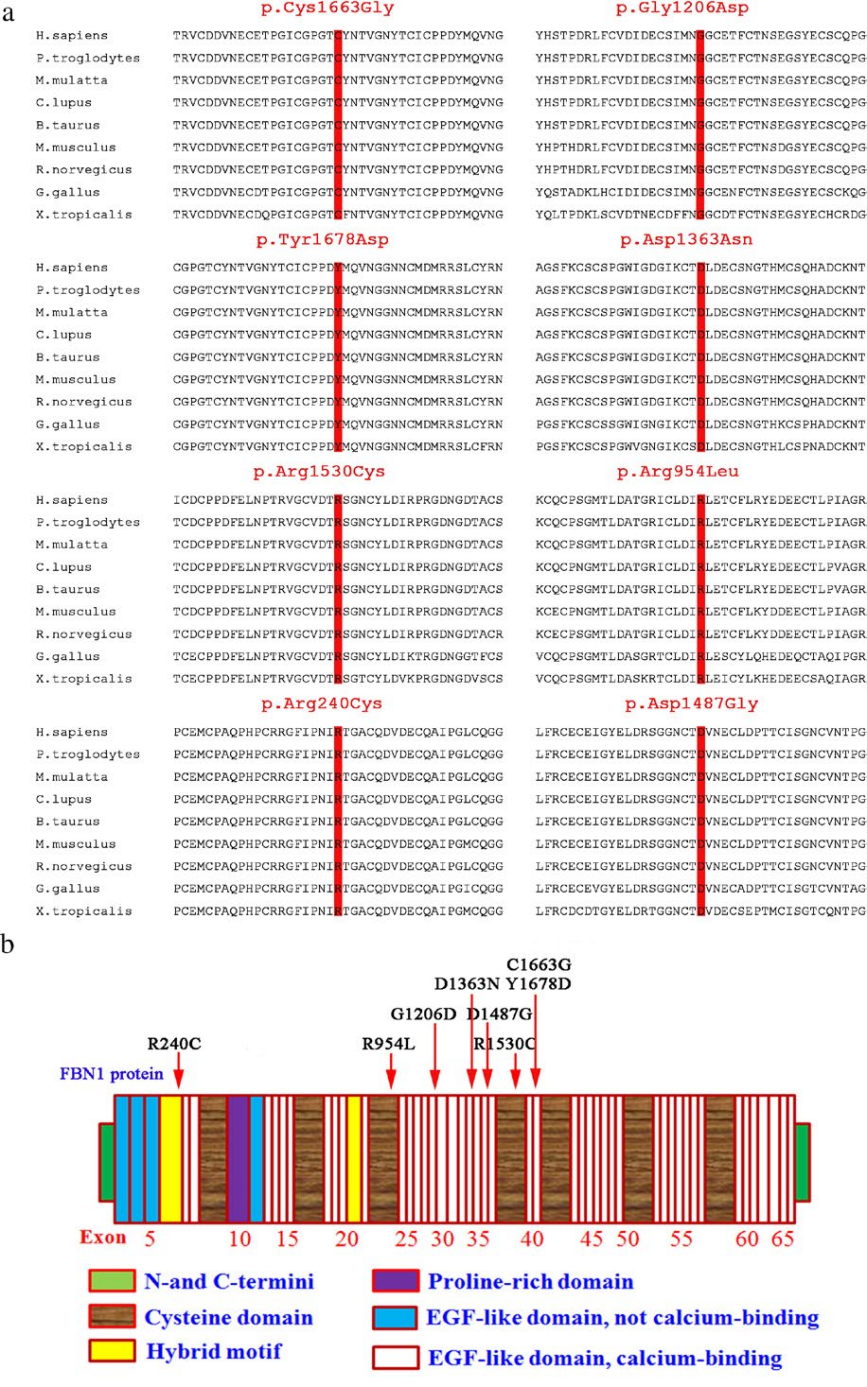
Orthologous protein sequence alignment and structure diagram of FBN1 sequence. (a) Orthologous protein sequence alignment showed the eight mutations sites happened in a highly conserved region of FBN1 among different species; (b) six mutations occurred in the calcium binding EGF‐like domain and two mutations happened in the cysteine domain of FBN1

Comparative amino acid sequence alignment of FBN1 protein across different species, including *Pan  troglodytes*, *Macaca  mulatta*, *Canis  lupus*, *Bos  taurus*, *Mus  musculus*, *Rattus  norvegicus*, *Gallus  gallus*, and *Xenopus  tropicalis*, revealed that these eight mutations happened in a highly conserved region of FBN1 (Figure 3b). Protein structure prediction showed that the majority of mutations (5/8) including c.4987T>G, c.3617G>A, c.5032T>G, c.4087G>A, and c.4460 A>G located in a highly conserved region of the calcium binding epidermal growth factor‐like (cbEGF) domain, two mutations c.2861G>T (p.R954L) and c.4588C>T (p.R1530C) occurred in the cysteine domain, the rest one mutation c.718C>T (p.R240C) occurred in the hybird module domain (Figure 4b and Table 2). These amino acid substitutions in the fibrillin‐1 was predicted to be deleterious, probably damaging and disease causing by SIFT, PolyPhen‑2, Mutationtaster, and ACMG classification. (Table 2), respectively.

## 4 DISCUSSION

MFS is an autosomal dominant disorder of connective tissue abnormality typically involving the ocular, skeletal, and cardiovascular systems (Judge & Dietz, 2005). The incidence of MFS is 1/10,000–2/10,000 (Groth et al., 2015), and more than 20% are sporadic cases (Judge & Dietz, 2005). Previous studies showed that MFS is mainly caused by mutations in the *FBN1* gene (Li et al., 2012). The reported *FBN1* mutations mainly include substitution, deletion, insertion, and duplication. In this study, we identified eight heterozygous mutations, including four novel mutations and four known mutations in the *FBN1* gene from eight pedigrees.

Fibrillin‐1 is a vital element of microfibrils and exists in many human tissues, such as tendon, cornea, zonules, cartilage, the cardiovascular system, and so on (Wang, Li, Lan, & Li, 2015). It is mainly comprised of repeated modules such as cbEGF domains and cysteine domains (Sakai, 1986). CbEGF is responsible for maintaining microfibers in an ordered arrangement (Dietz et al., 1991). It was reported that the most common type is a missense mutation and most mutations of *FBN1* occur in the cbEGF‐like domains which would induce a critical functional change of the domain itself and neighboring domains (Dietz, Saraiva, Pyeritz, Cutting, & Francomano, 2010), and the majority of mutations are cysteine substitutions (Yang et al., 2016). In addition, each of the EGF‐like domains contains six highly conserved cysteine residues EGF that form three disulfide bridges. These bridges enhance calcium binding; seven transforming growth factor β binding protein‐like modules (8‐Cys/TB). Each 8‐Cys/TB module is characterized by eight highly conserved cysteine residues, which serve to hold TGF‐β in an inactive complex (Jin et al., 2007). In our studies, all detected mutations are missense heterozygous mutation and six of eight located within the cbEGF‐like domains and two of the eight located within cysteine domain. Theses eight mutations may cause disrupt abnormal formation of microfibrils, thereby resulting in incorrect function FBN1 protein.

The mutation of *FBN1* has been reported to cause many complications (Judge & Dietz, 2005). In family 1, ectopia lentis was observed in both eyes of the proband and her young sisters (III:2 and III:3) who carry the mutation c.4987T>G (p.C1663G). In addition, all the affected members in this family had aortic aneurysm. The mutation c.718C>T (p.R240C) in *FBN1*, identified in family 7, was first reported in 2001 (Loeys, Nuytinck, Delvaux, De, & De, 2001) in the patient who had ectopia lentis and mild cardiovascular manifestations. Affected members carrying this mutation also were found to have cardiovascular and ocular characteristic of MFS with normal skeletal system in three large Hispanic families from Mexico (Villamizar et al., 2010). A Chinese MFS patient with this mutation was reported to have ocular and skeletal abnormalities (Jin et al., 2007). The mutation c.4588C>T (p.R1530C), which had been reported to be related to MFS in previous study (Collodbéroud et al., 1998), was found in the proband and other affected MFS patients in family 5. The mutation c.4987T>G (p.C1663G), similar to c.4987T>C (p.C1663R) (Dietz et al., 1991), caused a deficient cysteine residue within the cbEGF‐like module. This change could result in module misfolding and may have deleterious effects on the global structure of fibrillin‐1. The mutations c.4460A>G (p.D1487G) and c.3617G>A (p.G1206D) were reported in ClinVar database and the former one is similar to the reported mutation c.4460A>C (p.D1487A) (Whiteman & Handford, 2003), which may cause disruption of ligand binding site and delay intracellular processing and/or secretion from the cell that lead to severe reduction of matrix deposition and development of MFS.

Combined with the clinical data, the results showed that these novel mutations may play an important role in the pathogenesis of MFS development. To date, there is evidence that most of the *FBN1* mutations are clustered in exons 24–32, a hot spot region associated with classic and severe type or neonatal type of MFS (Whiteman & Handford, 2003). It is worth noting that the majority of patients with cysteine substitutions have classical MFS, and cysteine substitutions in exons 26–32 appear to be associated with classical disease manifesting early in life (Faivre et al., 2007). Interestingly, mutations in exons 12–15 encoding cbEGF‐like domains (C3‐C6) usually cause a mild MFS with possible late cardiovascular involvement. Most strikingly, Rommel et al. (2010) also showed a significantly higher incidence of ectopia lentis in patients who carried a mutations involved a cysteine substitution, as compared to patients whose mutation that led to a missense mutation without cysteine involvement in FBN1. In the study, clinical data from eight pedigrees showed that these mutations were responsible for MFS and related disorder in Chinese families. However, we need further functional analyses to confirm the role of fibrillin‐1 and its underlying mechanisms in MFS.

## 5 CONCLUSION

Four novel mutations and four known heterozygous mutations in the *FBN1* gene from eight Chinese families were identified to be associated with MFS and related disorder. Our data further enriches the *FBN1* mutation spectrum and may shed light on the pathogenesis, clinical diagnosis and management of MFS.

## CONFLICT OF INTERESTS

None declared.
